# Diagnosing and grading gastric atrophy and intestinal metaplasia using semi-supervised deep learning on pathological images: development and validation study

**DOI:** 10.1007/s10120-023-01451-9

**Published:** 2023-12-14

**Authors:** Shuangshuang Fang, Zhenyu Liu, Qi Qiu, Zhenchao Tang, Yang Yang, Zhongsheng Kuang, Xiaohua Du, Shanshan Xiao, Yanyan Liu, Yuanbin Luo, Liping Gu, Li Tian, Xiaoxia Liang, Guiling Fan, Yu Zhang, Ping Zhang, Weixun Zhou, Xiuli Liu, Jie Tian, Wei Wei

**Affiliations:** 1 Beijing Key Laboratory of Functional Gastrointestinal Disorders Diagnosis and Treatment of Traditional Chinese Medicine; Department of Gastroenterology, Wangjing Hospital, China Academy of Chinese Medical Sciences, No. 6, Zhonghuan South Road, Wangjing, Beijing, 100102 China; 2 CAS Key Laboratory of Molecular Imaging, Institute of Automation, Chinese Academy of Sciences, Beijing, 100190 China; 3 School of Artificial Intelligence, University of Chinese Academy of Science, Beijing, 100190 China; 4 Beijing Advanced Innovation Center for Big Data-Based Precision Medicine, School of Engineering Medicine, Beihang University, Beijing, 100191 China; 5 Department of Pathology, The First Affiliated Hospital of Guangdong University of Traditional Chinese Medicine, Guangzhou, 510405 China; 6 Department of Pathology, Guangdong Provincial Hospital of Traditional Chinese Medicine, Guangzhou, 510120 China; 7 Department of Pathology, Gansu Provincial Hospital of Traditional Chinese Medicine, Lanzhou, 730050 China; 8 Department of Pathology, Shanxi Provincial Hospital of Traditional Chinese Medicine, Taiyuan, 030012 China; 9 Department of Pathology, Wangjing Hospital, China Academy of Chinese Medical Sciences, Beijing, 100102 China; 10 Department of Pathology, Peking Union Medical College Hospital, Beijing, 100730 China; 11 Department of Pathology and Immunology, Washington University, St. Louis, MO 98195 USA; 12 Engineering Research Center of Molecular and Neuro Imaging of Ministry of Education, School of Life Science and Technology, Xidian University, Xi’an, 710126 Shaanxi China

**Keywords:** Atrophic gastritis, Semi-supervised deep learning, Diagnose, The operative link for gastric intestinal metaplasia assessment, The operative link for gastritis assessment

## Abstract

**Objective:**

Patients with gastric atrophy and intestinal metaplasia (IM) were at risk for gastric cancer, necessitating an accurate risk assessment. We aimed to establish and validate a diagnostic approach for gastric biopsy specimens using deep learning and OLGA/OLGIM for individual gastric cancer risk classification.

**Methods:**

In this study, we prospectively enrolled 545 patients suspected of atrophic gastritis during endoscopy from 13 tertiary hospitals between December 22, 2017, to September 25, 2020, with a total of 2725 whole-slide images (WSIs). Patients were randomly divided into a training set (*n* = 349), an internal validation set (*n* = 87), and an external validation set (*n* = 109). Sixty patients from the external validation set were randomly selected and divided into two groups for an observer study, one with the assistance of algorithm results and the other without. We proposed a semi-supervised deep learning algorithm to diagnose and grade IM and atrophy, and we compared it with the assessments of 10 pathologists. The model’s performance was evaluated based on the area under the curve (AUC), sensitivity, specificity, and weighted kappa value.

**Results:**

The algorithm, named GasMIL, was established and demonstrated encouraging performance in diagnosing IM (AUC 0.884, 95% CI 0.862–0.902) and atrophy (AUC 0.877, 95% CI 0.855–0.897) in the external test set. In the observer study, GasMIL achieved an 80% sensitivity, 85% specificity, a weighted kappa value of 0.61, and an AUC of 0.953, surpassing the performance of all ten pathologists in diagnosing atrophy. Among the 10 pathologists, GasMIL’s AUC ranked second in OLGA (0.729, 95% CI 0.625–0.833) and fifth in OLGIM (0.792, 95% CI 0.688–0.896). With the assistance of GasMIL, pathologists demonstrated improved AUC (*p* = 0.013), sensitivity (*p* = 0.014), and weighted kappa (*p* = 0.016) in diagnosing IM, and improved specificity (*p* = 0.007) in diagnosing atrophy compared to pathologists working alone.

**Conclusion:**

GasMIL shows the best overall performance in diagnosing IM and atrophy when compared to pathologists, significantly enhancing their diagnostic capabilities.

**Supplementary Information:**

The online version contains supplementary material available at 10.1007/s10120-023-01451-9.

## Introduction

Gastric cancer (GC) is a major global health concern, ranking as the fifth most commonly diagnosed malignant tumor and the fourth leading cause of cancer-related deaths worldwide [1]. More than 95% of GC are adenocarcinomas [2], of which intestinal adenocarcinoma is the most common type [3].

Several pathologies of chronic atrophic gastritis (CAG), including atrophy, intestinal metaplasia (IM), and dysplasia, are important pathways for the development of intestinal-type adenocarcinoma from normal mucosa (also known as the “Correa cascade”) [4]. The GC risk in CAG patients gradually increases with the progression of the Correa cascade. Recent studies [5, 6] have shown that the annual incidence rates of gastric cancer for atrophy, intestinal metaplasia, and dysplasia are 0.1%, 0.12–0.25%, and 0.6–6%, respectively. The operative link for gastric intestinal metaplasia assessment (OLGIM) [7] and operative link for gastritis assessment (OLGA) [8] by integrating the IM/atrophy score and the topography [9] have been advocated by international guidelines [3, 10, 11] for risk stratification of individuals diagnosed with gastric precancerous conditions. However, accurately assessing pathological IM and atrophy and stratifying OLGIM/OLGA risk have been difficult for pathologists.

Since the Sydney system was updated in 1994 [12], pathologists have continuously pointed out that the diagnostic system can be challenging in clinical practice [5]. The diagnosis of atrophic gastritis needs grading the severity of gland loss, which is difficult to evaluate quantitatively with accuracy, resulting in poor precision [11]. To overcome this difficulty, pathologists have tried various methods, including holding meetings to unify conceptual terminology [13], cycling a group of pathological pictures for repeated training [14], and proposing some intuitive measurement methods [15], etc. However, these efforts have failed to effectively improve accuracy, or the methods were difficult to apply widely. So far, the consistency and accuracy of pathological histology in patients with CAG for diagnosis and GC risk stratification remain limited [3, 6, 10, 11].

Deep learning has shown potential in medical image analysis. Automatic recognition technology based on deep learning has also achieved outstanding results in the diagnosis of digital pathological images, such as breast cancer, lung cancer, colorectal cancer, and prostate cancer [16–21]. Under certain conditions, the diagnostic performance of these artificial intelligence models is not inferior to that of human experts. However, these studies often use fully supervised learning, requiring pathologists to manually label lesions for pixel-level training, which is easily affected by various factors. Deep learning based on weak supervision can automatically mine suspicious lesions only with accurate category information. It is suitable for situations that are highly affected by subjective factors or difficult to obtain manual annotations. It is expected to be applied to computational pathology to further improve the accuracy and consistency of diagnosis [22–24].

In this study, 2725 whole-slide images (WSIs) were collected continuously from 545 endoscopic suspected CAG patients in a multi-center trial to establish a deep neural network-based diagnostic model named GasMIL. The randomized observer study was conducted to verify the accuracy and consistency of the diagnoses made by pathologists assisted with GasMIL. We aimed to establish and validate a convolutional neural network algorithm to diagnose and risk stratification of individuals diagnosed with precancerous gastric mucosal changes.

## Methods

### Study design

**Fig. 1:**
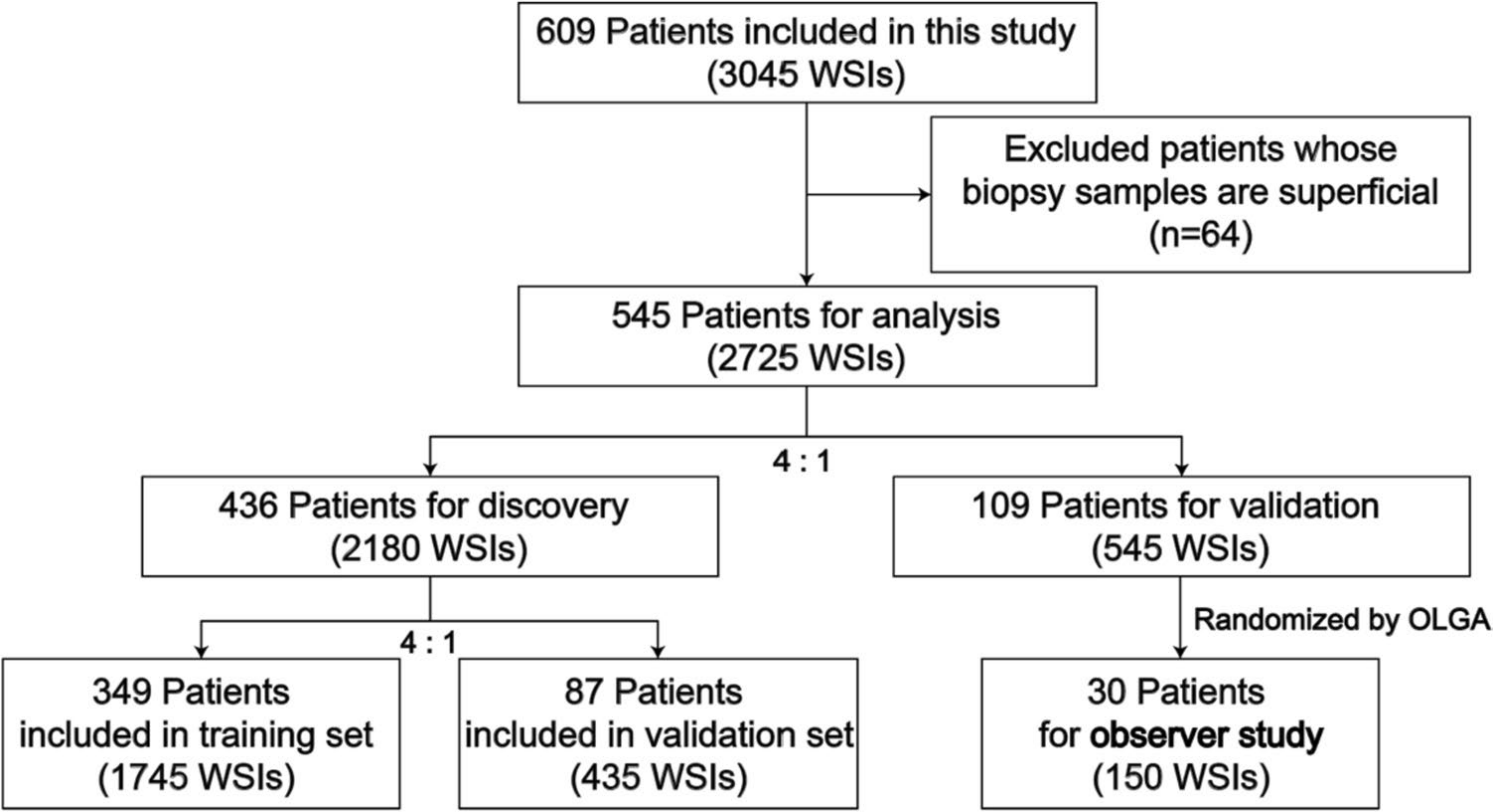
Study flowchart

The data sets of this study were obtained from a multicentre, prospective trial registered at https://clinicaltrials.gov/ (register number: NCT02955134). All authors had access to the study data, reviewed, and approved the final manuscript. From December 22, 2017, to September 25, 2020, 545 patients suspected of having atrophic gastritis were consecutively included during endoscopy at 13 tertiary hospitals. According to a 4:1 ratio, patients were randomly divided into a model construction set and a test set. The part used for model construction was further randomly divided into a training set and a validation set according to a ratio of 4:1 (Fig. 1).

The ethics committees of the 13 tertiary hospitals approved the trial protocol, and all participants signed informed consent forms. An independent data safety monitoring committee was responsible for monitoring the progress and safety of the trial.

### Participants and biopsy assessment

The inclusion criteria were patients aged 40–65 years suspected of having chronic atrophic gastritis during endoscopy. The exclusion criteria included autoimmune gastritis, gastric or duodenal ulcers, upper gastrointestinal bleeding, high-grade intraepithelial neoplasia in the gastric mucosa, or suspected malignant transformation based on histological diagnosis.

For patients meeting the inclusion and exclusion criteria, specimens were taken from 5 sites in the stomach, including two from the lesser and the greater curvature of the antrum (both within 2–3 cm from the pylorus), one from the lesser curvature of the corpus (about 4 cm proximal to the angulus), one from the middle portion of the greater curvature of the corpus (approximately 8 cm from the cardia), and one from the incisura angularis. The tissues were sliced, scanned using MoticEasyScan Pro, and then uploaded to the online diagnostic system.

WSIs were reviewed by two pathologists, and the superficial slices were removed for the follow-up study. Three experienced pathologists independently graded diagnoses according to the New Sydney system [12] (see Supplementary Figure S1 for diagnostic criteria), and the final diagnosis result was obtained after discussing any inconsistencies.

### Model development

**Fig. 2:**
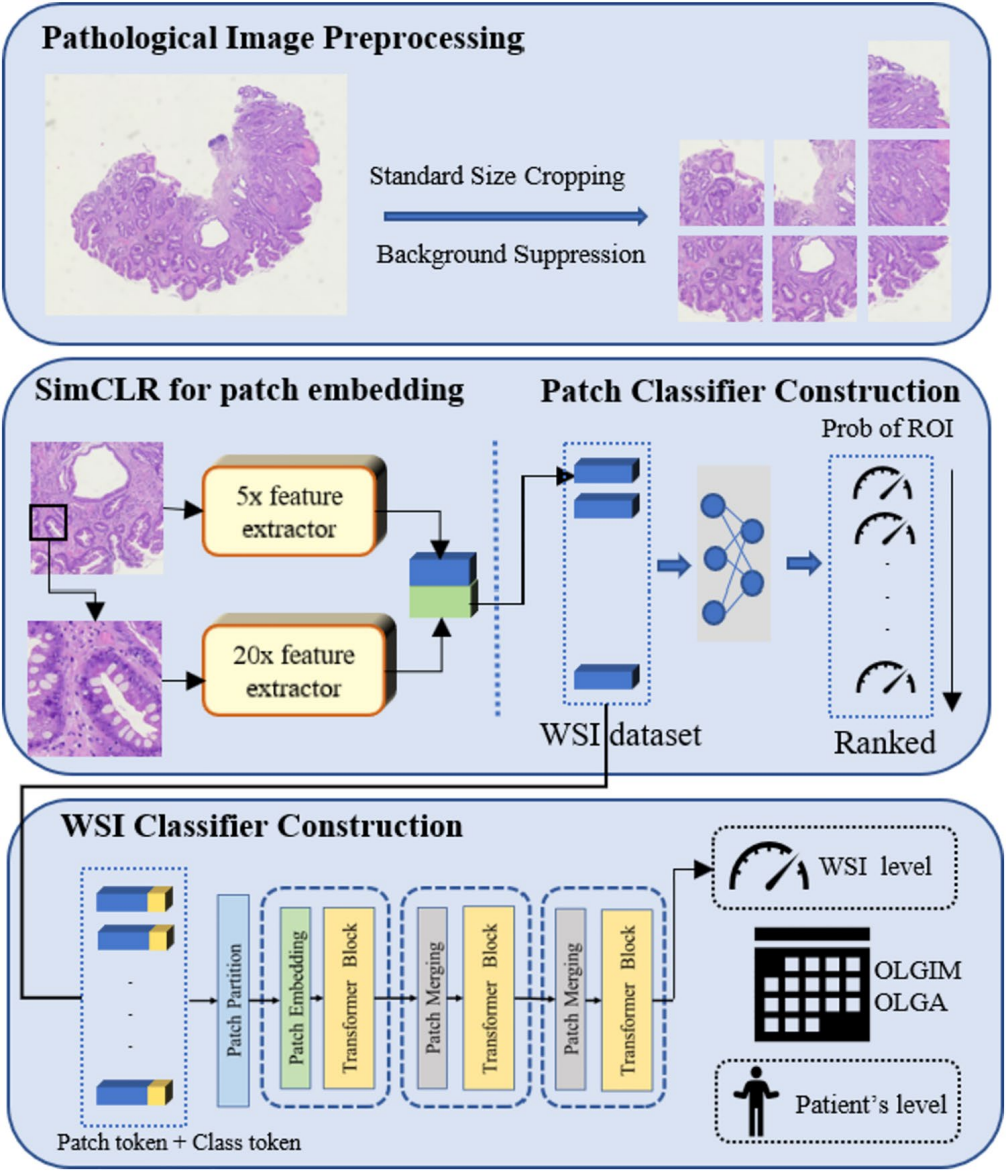
Workflow for the prognostic analysis of atrophic gastritis with deep learning

We proposed a deep neural network named GasMIL (Fig. 2) to predict the degree of atrophy/IM of gastric tissue slice images. Based on the pathological slice images from five parts of each patient, a patient-level grading prediction was comprehensively obtained. We apply weakly supervised learning in our algorithmic framework, specifically multiple instance learning (MIL). Unlike traditional deep convolutional neural networks, MIL only requires coarse-grained labels for the pathological diagnosis of each image, avoiding the need for complicated manual annotations by doctors. Additionally, we constructed MIL features for pathological images of different resolutions and performed multi-scale aggregation. The basic principle of this design is that shallow low-level features (such as local edges and textures) and deep high-level features (such as severe disease appearance) contain useful information for grade prediction and extracting and integrating comprehensive multi-scale image features helps in making the best grade decision.

#### Self-supervised learning for learning patch embedding

We cropped each WSI into non-overlapping blocks at resolutions of 224 × 224 with a field of view of 0.5 µm per pixel (MPP) and 2.0 MPP, respectively. Before multi-instance learning, we pre-learn embedding for each cut patch using SimCLR [25] proposed by “Hinton’s team”. This simple framework for contrastive learning was employed to learn robust image representations without manual labeling. For each original patch, we applied several data augmentation operations (including random rotation, random color distortion, etc.) to generate sub-images and performed feature extraction through a resnet18-based encoder. We then constructed a contrastive loss to minimize the distance between these sub-images from the same original image in feature space. The output of the trained encoder was used for downstream MIL tasks.

#### Construction of multi-scale patch embedding

For both 0.5 MPP and 2.0 MPP magnifications, we constructed single-scale WSI classifiers. The patch embedding under 2.0 MPP magnification was spliced with the embedding corresponding to the physical 0.5 MPP magnification position to obtain a comprehensive patch embedding.

#### Using WSI classifier to select key patches

In the MIL hypothesis, when a WSI is marked as positive (label > 0), at least one patch is the target lesion area; if the mark is negative, all patch labels should be negative. Based on this assumption, we used the MLP network as a classifier to feed multi-scale patch embeddings. After completing the training of the patch-level classifier, we could obtain the probability of all patches in the current WSI being predicted as lesion areas and sort them to obtain the patches that should be the most focused on. We uniformly selected the top 20 patches with the highest ranking for each WSI to input into the downstream aggregator.

#### Apply the transformer to the aggregation of patches

The traditional MIL uses pooling algorithms to comprehensively evaluate the prediction probability of the top patches and obtain the prediction degree of WSI. However, these CV-based pooling algorithms ignore the correlation information between patches. Considering the potential model enhancements from these correlations, we introduced the transformer into the aggregation stage. The 100 most critical (most likely to be lesion area) patch vectors obtained from each WSI through the first step were sequentially passed to the transformer classifier to predict the entire rank probability of WSI.

#### Patient-level prediction model construction

For the WSI of five gastric parts in the same patient, we obtained five prediction grades through the WSI-level prediction model. According to the OLGA and OLGIM, we could then obtain the final patient-level prediction grade.

### Observer study

Sixty patients from the test set were randomly selected for observational studies. All clinical information was concealed and randomly divided into two groups: one with the aid of GasMIL diagnostic results and the other without. The digital WSIs were distributed to 10 pathologists for diagnosis, and the diagnosis results were recorded. The AUC, sensitivity, specificity, and consistency of the two groups of slides diagnosed by 10 pathologists were obtained.

### Statistical analysis

We employed a combination of statistical tests, including the T-test and Wilcoxon signed-rank test, to examine the impact of age at baseline and a combination of the Chi-square test, Fisher’s exact test, continuously corrected Chi-square test, and signed-rank test to analyze gender. The Wilcoxon signed-rank test was also used to compare baseline histological data.

Receiver operating characteristic (ROC) curves and area under the curve (AUC) were analyzed using the machine learning Python package sci-kit-learn to quantify diagnostic classifier performance, as well as accuracy, sensitivity, and specificity. The cutoff value of the ROC curve was set at 0.5. Cohen’s kappa coefficient was used to assess interobserver agreement between diagnostic models and human pathologists. Python and Pytorch were used to build WSI algorithms.

## Results

### Baseline characteristics

Between December 2017 and September 2020, up to 609 potentially eligible patients from 13 Chinese tertiary hospitals were enrolled in this study. Among them, 64 patients were excluded because the biopsy samples were superficial. Thus, 545 patients with 2725 WSIs were finally enrolled for analysis (Fig. 1).

After randomization of these patients, 349 patients with 1745 WSIs were assigned to the training set, 87 patients with 435 WSIs were assigned to the validation set, and the other 109 patients with 545 WSIs composed the test set. Their characteristics are summarized in Table 1. There were no significant differences in all baseline characteristics (*p* > 0.05) or the distribution of patients among OLGA and OLGIM stages (*p* > 0.05) between the training, validation, and test sets 2).

**Table 1:** Baseline characters of included patients

Characteristics	All patients (*N* = 546)	Training set (*N* = 348)	Validation set (*N* = 88)	Test set (*N* = 110)	*p* value
Age (years)	54.20 ± 9.73	54.21 ± 9.38	54.19 ± 10.81	54.08 ± 9.90	0.993
Female (%)	252 (46.15%)	157 (45.11%)	45 (51.14%)	50 (45.45%)	0.953
OLGA (%)					0.999
0	125 (22.89%)	80 (22.99%)	20 (22.73%)	25 (22.73%)	
1	137 (25.09%)	87 (25.00%)	22 (25.00%)	28 (25.45%)	
2	104 (19.05%)	66 (18.97%)	17 (19.32%)	21 (19.09%)	
3	111 (20.33%)	71 (20.40%)	18 (20.45%)	22 (20.00%)	
4	69 (12.64%)	44 (12.64%)	11 (12.50%)	14 (12.73%)	
OLGIM (%)					0.852
0	119 (21.79%)	79 (22.70%)	20 (22.73%)	20 (18.18%)	
1	136 (24.91%)	82 (23.56%)	23 (26.14%)	31 (28.18%)	
2	101 (18.50%)	67 (19.25%)	15 (17.05%)	19 (17.27%)	
3	118 (21.61%)	74 (21.26%)	19 (21.59%)	25 (22.73%)	
4	72 (13.19%)	46 (13.22%)	11 (12.50%)	15 (13.64%)

### Performance of the model on the training, validation, and test set

In the diagnostic model we built, the data set has a total of 546 patients and was randomly divided. To avoid model prediction bias caused by differences in the data distribution of each cohort, we used stratified sampling to divide the data set according to the true atrophy grade of the patients. We split patients into a training cohort and an external testing cohort in a ratio of 4:1. In the training cohort, 20% of the patients were used for internal validation of the model.

**Fig. 3:**
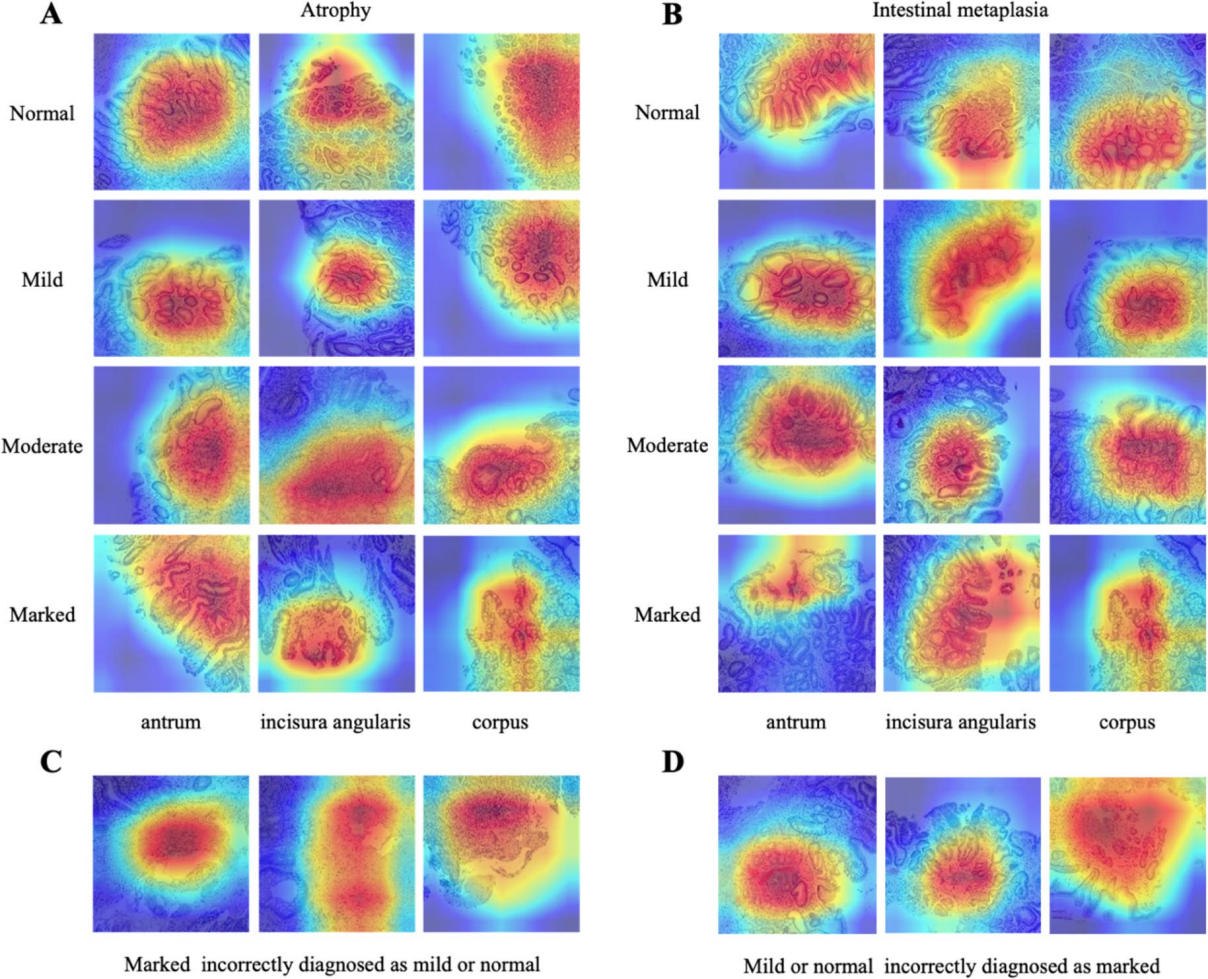
The representative histologic images, including both successful inferences and failures. **A** Representative heat map images with varying atrophy grades. **B** Representative heat map images with different intestinal metaplasia grades. **C** Instances of misdiagnosis by the GasMIL (marked incorrectly diagnosed as mild or normal). **D** Instances of misdiagnosis by the GasMIL (mild or normal incorrectly diagnosed as marked)

We use AUC to measure the ability of GasMIL to predict pathological images of different grades. Table 2 shows the model’s AUC, sensitivity, and specificity in the training, internal validation, and independent test cohorts. At the WSI level, GasMIL achieves good diagnostic performance in the independent test cohort (AUC_Inflammation_: 0.970; AUC_Activity_: 0.981; AUC_IM_: 0.884; AUC_Atrophy_: 0.877). The representative heat map images with varying atrophy/IM grades are shown in Fig. 3A and B. For a certain problem, we referred to OLGA and OLGIM to convert the WSI prediction grade for different stomachs of the same patient to the final prediction grade for that patient. At the patient level, the good predictive performance of the diagnostic model carried over (AUC_OLGIM_: 0.792; AUC_OLGA_: 0.729).

**Table 2:** Performance of the GasMIL on the training, validation, and test set

	*n*	AUC	Sensitivity	Specificity	Weighted kappa
Inflammation (slides)					
Training	1740	0.965 [0.959, 0.970]	0.86 [0.84, 0.88]	0.86 [0.84, 0.88]	0.65 [0.61, 0.68]
Validation	440	0.967 [0.955, 0.978]	0.87 [0.84, 0.90]	0.87 [0.84, 0.90]	0.67 [0.59, 0.73]
Test	550	0.970 [0.959, 0.980]	0.86 [0.83, 0.89]	0.86 [0.83, 0.89]	0.58 [0.50, 0.65]
Activity (slides)					
Training	1740	0.982 [0.978, 0.985]	0.87 [0.85, 0.88]	0.87 [0.85, 0.88]	0.48 [0.42, 0.54]
Validation	440	0.987 [0.980, 0.992]	0.90 [0.87, 0.93]	0.90 [0.87, 0.93]	0.43 [0.29, 0.57]
Test	550	0.981 [0.973, 0.988]	0.90 [0.87, 0.92]	0.90 [0.87, 0.92]	0.48 [0.36, 0.60]
IM (slides)					
Training	1740	0.915 [0.906, 0.924]	0.74 [0.72, 0.76]	0.74 [0.72, 0.76]	0.80 [0.77, 0.82]
Validation	440	0.912 [0.895, 0.930]	0.77 [0.73, 0.81]	0.77 [0.73, 0.81]	0.82 [0.77, 0.87]
Test	550	0.884 [0.862, 0.902]	0.69 [0.65, 0.72]	0.69 [0.65, 0.72]	0.70 [0.63, 0.76]
Atrophy (slides)					
Training	1740	0.914 [0.904, 0.922]	0.83 [0.82, 0.85]	0.83 [0.82, 0.85]	0.88 [0.86, 0.90]
Validation	440	0.919 [0.899, 0.936]	0.84 [0.80, 0.87]	0.84 [0.80, 0.87]	0.89 [0.85, 0.92]
Test	550	0.877 [0.855, 0.897]	0.70 [0.66, 0.74]	0.70 [0.66, 0.74]	0.62 [0.54, 0.68]
OLGIM					
Training	348	0.829 [0.799, 0.858]	0.73 [0.68, 0.77]	0.73 [0.68, 0.77]	0.88 [0.85, 0.91]
Validation	88	0.830 [0.773, 0.886]	0.73 [0.64, 0.82]	0.73 [0.62, 0.81]	0.88 [0.81, 0.93]
Test	110	0.790 [0.733, 0.841]	0.66 [0.57, 0.75]	0.66 [0.57, 0.75]	0.81 [0.72, 0.88]
OLGA					
Training	348	0.829 [0.801, 0.860]	0.73 [0.68, 0.78]	0.73 [0.68, 0.78]	0.88 [0.84, 0.91]
Validation	88	0.830 [0.773, 0.886]	0.73 [0.62, 0.82]	0.73 [0.62, 0.82]	0.88 [0.80, 0.93]
Test	110	0.790 [0.727, 0.847]	0.66 [0.57, 0.75]	0.66 [0.57, 0.75]	0.85 [0.78, 0.90]

As demonstrated in the cross-tabulation of GasMIL results with the gold standard (see Figure S1-4), among the 150 WSIs, two initially categorized as normal/mild atrophy were misdiagnosed as severe atrophy, and two initially labeled as severe atrophy were misdiagnosed as mild atrophy. Moreover, one case initially classified as normal was misdiagnosed as severe IM, and four initially categorized as severe IM were misdiagnosed as normal/mild IM. Retrieve the original image, we found that severe cases were often misdiagnosed as normal/mild due to significant inflammation and structural distortion (Fig. 3C). Additionally, when pathology sections intersected blood vessels or were excessively thin, normal/mild conditions were frequently misdiagnosed as severe (Fig. 3D).

### Results of the observer study compared with 10 pathologists

**Fig. 4:**
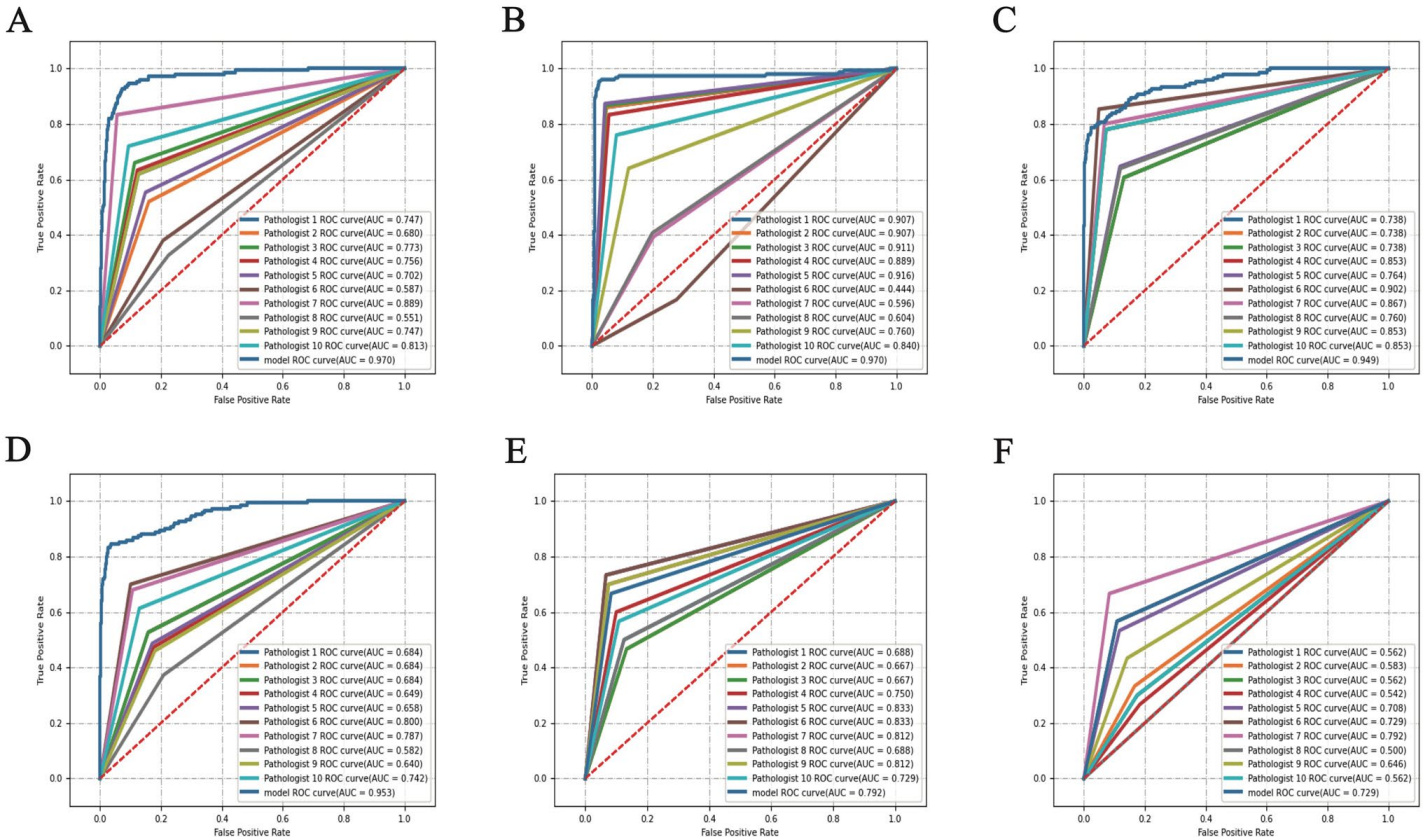
Comparison of receiver operating characteristic (ROC) curves between GasMIL and 10 pathologists. **A** Inflammation, **B** activity, **C** intestinal metaplasia, **D** atrophy; patient, **E** operative link for gastric intestinal metaplasia assessment (OLGIM), **F** operative link for gastritis assessment (OLGA)

The diagnostic results of GasMIL were compared with those of 10 pathologists on 30 patients with 150 WSIs. At the slide level, the ROC of the GasMIL for inflammation, activity, IM, and atrophy were 0.970 (95% CI 0.947–0.986), 0.970 (95% CI 0.941–0.998), 0.949 (95% CI 0.918–0.972) and 0.953 (95% CI 0.927–0.976), respectively (Fig. 4A–D), and were higher than those of 10 pathologists. The diagnostic sensitivity, specificity, and weighted kappa value of the GasMIL for inflammation, activity, IM, and atrophy were also higher than those of most pathologists (supplementary table S5).

The ROC for the OLGIM model was 0.792, which was higher than 6 out of 10 pathologists. For OLGA, GasMIL’s ROC was 0.729, ranking second among the 10 pathologists (Fig. 4E, F). The diagnostic sensitivity, specificity, and weighted kappa value of the artificial intelligence diagnostic model for OLGA and OLGIM are also higher than those of most pathologists (supplementary table S5).

### The results of GasMIL-assisted diagnosis compared with pathologist-independent diagnosis

**Fig. 5:**
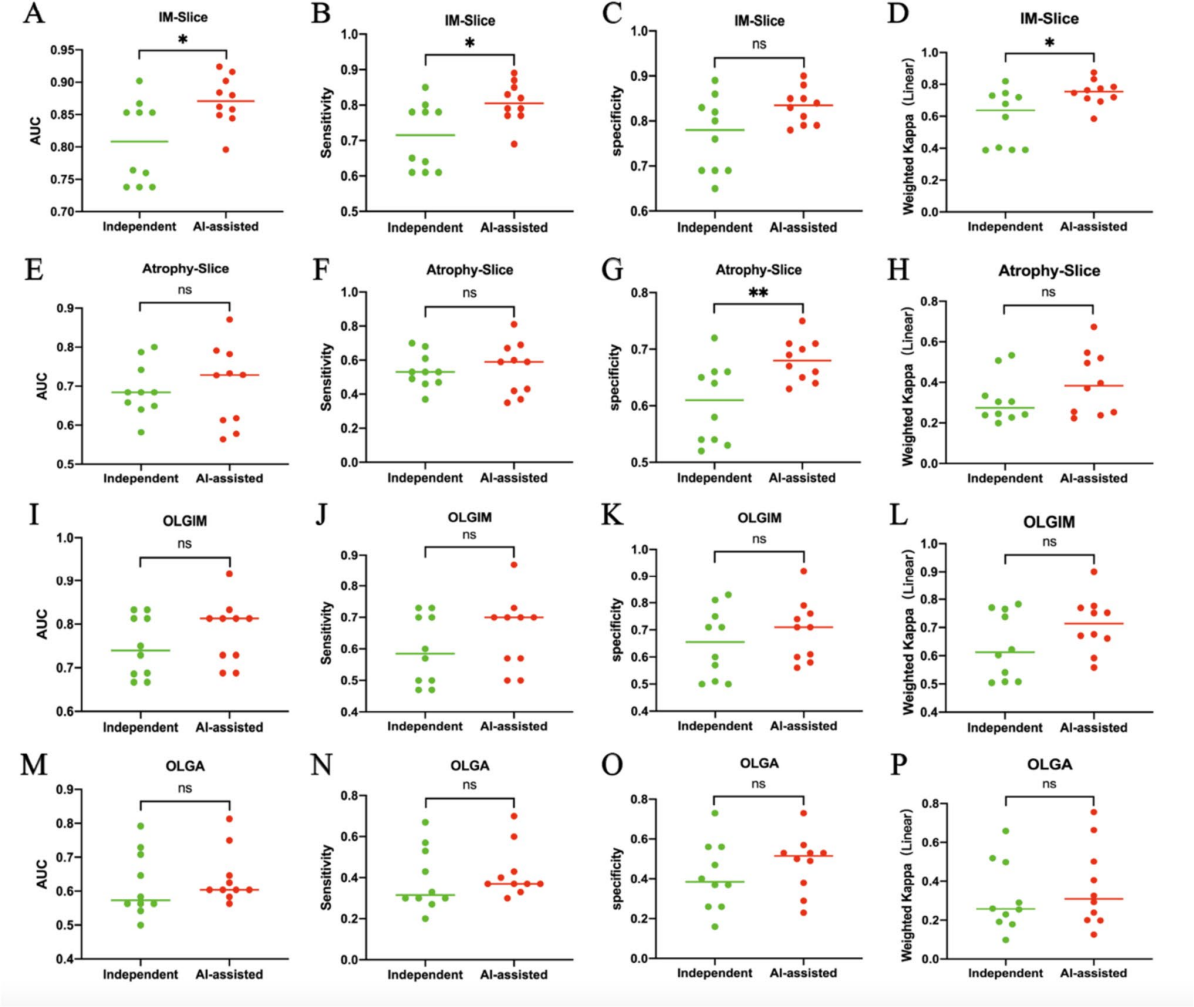
The scatterplot of GasMIL-assisted diagnosis compared with pathologist-independent diagnosis. **A**–**D** The area under the curve (AUC), sensitivity, specificity, and weighted kappa of the two groups of slides intestinal metaplasia was diagnosed by 10 pathologists. **E**–**H** Ten pathologists diagnosed the atrophy of two groups of slides. **I**–**L** Ten pathologists diagnosed the OLGIM of two groups of patients. **M**–**P** Ten pathologists diagnosed the OLGA of two groups of patients

We compared the results of pathologists with and without GasMIL assistance on two groups of 30 patients with 150 WSIs. For IM, pathologists have higher AUC, sensitivity, and weighted kappa (*p* = 0.013, 0.014, 0.016, respectively, Fig. 5A, B, D) with the assistance of GasMIL, but there was no statistical difference in specificity (*p* > 0.05, Fig. 5C). For atrophy, pathologists had higher specificity with the assistance of GasMIL (*p* = 0.007, Fig. 5G), but there was no statistical difference in AUC, sensitivity, and weighted kappa (*p* > 0.05, Fig. 5E, F, H).

At the patient level, whether pathologists were assisted with GasMIL or not, the AUC, sensitivity, specificity, and weighted kappa of OLGIM and OLGA showed no statistical difference (*p* > 0.05, Fig. 5I–P), but there was a trend of improvement.

## Discussion

Recent studies have shown that deep learning-based algorithms were promising in classifying and grading pathological lesions in digitized H&E slides [26, 27]. Regarding the poor diagnostic accuracy and increasing diagnostic workload of endoscopic biopsy specimens call for a high-performance algorithm with high sensitivity and specificity [28].

In this study, we developed an algorithm named GasMIL to diagnose inflammation, activity, atrophy, and IM in gastric biopsy specimens and demonstrated superb performance better than all ten pathologists. Especially concerning atrophy, the concept is represented by the discrepancy between the expected glands and what is actually observed at the histologic exam [29], which can be subjective and pathologists are most likely to be inconsistent with [30, 31]. Accurately diagnosing atrophy was considered crucial for the prevention of gastric cancer, as a study found that 37.2% of patients who developed gastric cancer had been diagnosed with indefinite atrophy previously [32]. In the present study, the GasMIL showed an 80% sensitivity, 85% specificity, and 0.61 weighted kappa value on the observer study, which was higher than that of pathologists trained through four rounds of reading (kappa = 0.46) [14]. Therefore, at the slide level, GasMIL has the potential to serve as a tool for supervising pathologists to process the sheer number of samples in limited clinical working hours.

To estimate whether the GasMIL model can accurately stratify GC risk in CAG patients as well, OLGIM and OLGA were obtained by combining the results of 5 WSIs. In the observer study, GasMIL showed the second-highest diagnostic accuracy in OLGA and fifth in OLGIM among the ten pathologists, with AUCs of 0.72 and 0.79, respectively. In contrast to OLGA, OLGIM reports a high interobserver concordance, consistent with our observer study [7, 33]. However, OLGIM staging was considered less sensitive than OLGA staging for it downgrades high-risk patients to low-risk groups [34]. Obtaining both OLGA and OLGIM information on the same pathological slide is beneficial for the secondary prevention of GC [9, 35]. At the patient level, high overall accuracy for the GasMIL in GC risk stratification was observed, suggesting that it can assist clinicians in individual GC risk stratification.

In addition, we conducted an observer study to investigate whether GasMIL can help pathologists improve diagnostic accuracy. The results revealed that with the assistance of GasMIL, the accuracy of pathologists in diagnosing IM significantly improved, as did the diagnostic specificity of atrophy. However, its accuracy in diagnosing OLGIM and OLGA did not significantly improve. Since the sample size analysis for the observer study was based on slides [36], the sample size of 30 patients may be too small to detect a statistical difference between OLGIM and OLGA. However, an increasing trend can be observed in Fig. 4 for their higher median with the assistance of GasMIL, and a follow-up observer study with a larger sample size is needed to evaluate its role in helping diagnose OLGIM and OLGA.

To the best of our knowledge, this is the first study that aimed to establish and validate a convolutional neural network algorithm to diagnose and grade OLGA and OLGIM based on the updated Sydney protocol. The AGA [3] recommends that gastric biopsies according to the updated Sydney system should become standard in the diagnostic workup for dyspepsia and gastritis, a step that has been shown to increase the detection rate of H. pylori and IM [37]. After a standard biopsy, it is essential to ensure that the pathologist has histologic scoring of gastric biopsy (for OLGA/OLGIM staging), avoiding the possibility of a secondary staging exam to determine risk level. However, in the grading of the biopsy IM and atrophy, many pathologists worldwide do not report severity scoring routinely [38]. The possible reason is that the judgment of severity scoring is quite subjective based on the proportion, making it difficult for pathologists to give an accurate and consistent result [13, 39, 40]. Therefore, we built a classification model for atrophy and IM using artificial intelligence techniques that can help quantify severity scores. High-quality multi-center data with strict quality control of all image acquisitions and histological analysis for every individual were used in this study, and a reliable gold standard diagnosis was jointly made by three experienced pathologists. Furthermore, GasMIL was fully validated, including the test set validation, compared with ten pathologists, and GasMIL auxiliary diagnosis compared with pathologists alone to investigate the robustness and reliability of GasMIL. The results proved that applying GasMIL for the quantitative analysis of WSIs offered valuable benefits for diagnosing atrophy and IM as well as GC risk in patients with CAG. Once the GasMIL model is established, pathologists only need to review and confirm the results of the model classification in the daily workflow, which is extremely easy for clinical applications.

Some studies have applied Convolutional Neuronal Networks technology to diagnose gastritis on biopsy H&E images. Panagiotis et al. reported a digital pathology framework for gastric gland segmentation and classification that achieved object dice scores equal to 0.908 and 0.967, respectively, in a dataset consisting of 20 patients with 85 WSIs of normal, gastric atrophy, and IM [41]. Georg et al. reported a convolutional neuronal network-based algorithm to classify gastritis into autoimmune, bacterial, and chemical subtypes, achieving an overall accuracy of 84% in a data set of 135 patients [42]. However, their digital pathology framework and study design were fundamentally different from ours. We focus on the problem that pathologists are having difficulty reaching a consensus when making diagnoses according to the updated Sydney system, we established an algorithm that can precisely grade the severity of atrophy and IM and can calculate the GC risk accordingly which is also beneficial to determine follow-up intervals.

Our study has some limitations. First, our model is independently developed and verified based on different gastric problems. This approach will increase machine memory overhead and does not further consider the relationship between image representations of different gastric problems. Secondly, as a black box model, the interpretability of the deep learning model is still poor. We tried to observe the model’s attention to different areas in the form of a heat map, but it is still difficult to explain how the model learns. Thirdly, the sample size of the observer study was insufficient for detecting the statistical difference between GasMIL auxiliary diagnosis and pathologists alone to diagnose OLGIM and OLGA, a larger sample size study is needed.

In conclusion, GasMIL shows the best overall performance in diagnosing inflammation, activity, IM, and atrophy, ranking fifth in diagnosing OLGIM and second in OLGA compared to ten pathologists. GasMIL-assisted significantly improves the performance of pathologists in diagnosing IM and atrophy. All of this suggested a clinical application potential of GasMIL for accurate pathological grading and GC risk stratification in atrophic gastritis patients.

### Supplementary Information

Below is the link to the electronic supplementary material.

Supplementary file1 (JPG 94 kb)

Supplementary file2 (DOCX 31 kb)

## Data Availability

All authors confirm that they have full access to all the data in the study and approved the final version of the article.
