# Supplementary figures and images for: Diagnosing and grading gastric atrophy and intestinal metaplasia using semi-supervised deep learning on pathological images: development and validation study
Source: Gastric Cancer. 2023 Dec 14;27(2):343–54. doi: 10.1007/s10120-023-01451-9 (PMC10896941; doi:10.1007/s10120-023-01451-9)

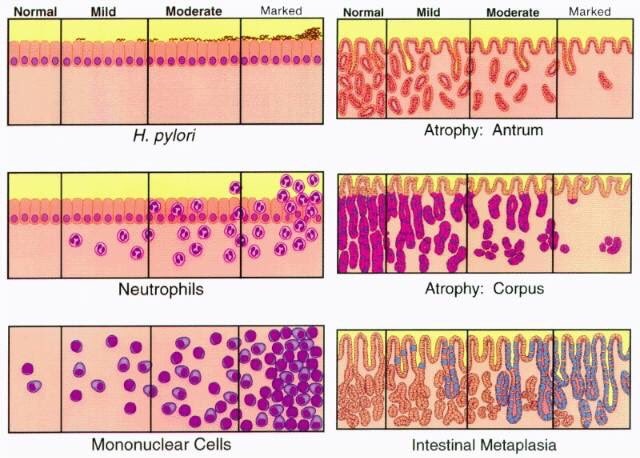

Supplement: Supplementary file 1 — Supplementary file1 (JPG 94 kb) [file 10120_2023_1451_MOESM1_ESM.jpg]
