# Supplementary material for: Diagnosing and grading gastric atrophy and intestinal metaplasia using semi-supervised deep learning on pathological images: development and validation study
Source: Gastric Cancer. 2023 Dec 14;27(2):343–54. doi: 10.1007/s10120-023-01451-9 (PMC10896941; doi:10.1007/s10120-023-01451-9)
Supplement: Supplementary file 2 — Supplementary file2 (DOCX 31 kb) [file 10120_2023_1451_MOESM2_ESM.docx]

| Atrophy | GasMIL predicted | | | | | Total |
| --- | --- | --- | --- | --- | --- | --- |
|  | Grade | 0 | 1 | 2 | 3 |  |
| Gold standard | 0 | 67 | 21 | 6 | 1 | 95 |
|  | 1 | 11 | 15 | 5 | 1 | 32 |
|  | 2 | 1 | 1 | 10 | 0 | 12 |
|  | 3 | 0 | 2 | 0 | 9 | 11 |
| Total | | 79 | 39 | 21 | 11 | 150 |

Table S1: Cross-tabulation of the GasMIL result compared to the gold standard in atrophy.

| IM | GasMIL predicted | | | | | Total |
| --- | --- | --- | --- | --- | --- | --- |
|  | Grade | 0 | 1 | 2 | 3 |  |
| Gold standard | 0 | 89 | 7 | 0 | 1 | 97 |
|  | 1 | 9 | 14 | 4 | 0 | 27 |
|  | 2 | 0 | 4 | 7 | 0 | 11 |
|  | 3 | 1 | 3 | 0 | 11 | 15 |
| Total | | 99 | 28 | 11 | 12 | 150 |

Table S2: Cross-tabulation of the GasMIL result compared to the gold standard in intestinal metaplasia.

| OLGA | GasMIL predicted | | | | | | Total |
| --- | --- | --- | --- | --- | --- | --- | --- |
|  | Grade | 0 | 1 | 2 | 3 | 4 |  |
| Gold standard | 0 | 7 | 0 | 0 | 0 | 0 | 7 |
|  | 1 | 0 | 5 | 1 | 2 | 0 | 8 |
|  | 2 | 0 | 4 | 1 | 3 | 0 | 8 |
|  | 3 | 0 | 2 | 0 | 2 | 0 | 4 |
|  | 4 | 0 | 0 | 0 | 1 | 2 | 3 |
| Total | | 7 | 11 | 2 | 8 | 2 | 30 |

Table S3: Cross-tabulation of the GasMIL result compared to the gold standard in OLGA.

| OLGIM | GasMIL predicted | | | | | | Total |
| --- | --- | --- | --- | --- | --- | --- | --- |
|  | Grade | 0 | 1 | 2 | 3 | 4 |  |
| Gold standard | 0 | 4 | 2 | 0 | 0 | 0 | 6 |
|  | 1 | 0 | 5 | 2 | 1 | 0 | 8 |
|  | 2 | 0 | 2 | 3 | 1 | 0 | 6 |
|  | 3 | 0 | 2 | 0 | 6 | 0 | 8 |
|  | 4 | 0 | 0 | 0 | 0 | 2 | 2 |
| Total | | 4 | 11 | 5 | 8 | 2 | 30 |

Table S4: Cross-tabulation of the GasMIL result compared to the gold standard in OLGIM.

Table S5 **Results of the observer study compared with 10 pathologists**

|  | AUC | Sensitivity | Specificity | Weighted kappa |
| --- | --- | --- | --- | --- |
| Inflammation (slides) |  |  |  |  |
| Pathologist 1 | 0.747[0.698,0.796] | 0.81[0.74,0.88] | 0.62[0.55,0.70] | 0.37[0.20,0.51] |
| Pathologist 2 | 0.680[0.627,0.738] | 0.86[0.79,0.92] | 0.52[0.45,0.60] | 0.38[0.22,0.51] |
| Pathologist 3 | 0.773[0.724,0.822] | 0.86[0.80,0.93] | 0.66[0.59,0.73] | 0.44[0.28,0.59] |
| Pathologist 4 | 0.756[0.707,0.804] | 0.85[0.80,0.89] | 0.63[0.55,0.71] | 0.47[0.35,0.57] |
| Pathologist 5 | 0.702[0.649,0.756] | 0.84[0.79,0.89] | 0.55[0.47,0.63] | 0.35[0.23,0.47] |
| Pathologist 6 | 0.587[0.538,0.640] | 0.82[0.77,0.87] | 0.38[0.30,0.45] | 0.27[0.18,0.36] |
| Pathologist 7 | 0.889[0.853,0.929] | 0.87[0.82,0.92] | 0.83[0.77,0.89] | 0.67[0.53,0.78] |
| Pathologist 8 | 0.551[0.502,0.600] | 0.82[0.76,0.87] | 0.33[0.26,0.41] | 0.24[0.15,0.32] |
| Pathologist 9 | 0.747[0.693,0.800] | 0.82[0.75,0.88] | 0.62[0.54,0.70] | 0.44[0.30,0.56] |
| Pathologist 10 | 0.813[0.764,0.858] | 0.84[0.79,0.89] | 0.72[0.65,0.79] | 0.48[0.35,0.61] |
| Model | 0.970[0.947,0.986] | 0.84[0.77,0.91] | 0.86[0.81,0.91] | 0.59[0.42,0.73] |
| Activity (slides) |  |  |  |  |
| Pathologist 1 | 0.907[0.871,0.942] | 0.86[0.79,0.91] | 0.86[0.80,0.91] | 0.49[0.22,0.69] |
| Pathologist 2 | 0.907[0.871,0.942] | 0.86[0.78,0.91] | 0.86[0.80,0.91] | 0.47[0.22,0.68] |
| Pathologist 3 | 0.911[0.875,0.942] | 0.87[0.80,0.92] | 0.87[0.81,0.92] | 0.50[0.24,0.72] |
| Pathologist 4 | 0.889[0.849,0.929] | 0.89[0.84,0.94] | 0.83[0.77,0.89] | 0.58[0.38,0.74] |
| Pathologist 5 | 0.916[0.876,0.947] | 0.84[0.76,0.91] | 0.87[0.82,0.92] | 0.31[0.10,0.52] |
| Pathologist 6 | 0.444[0.404,0.484] | 0.86[0.80,0.91] | 0.17[0.11,0.23] | 0.25[0.16,0.34] |
| Pathologist 7 | 0.596[0.542,0.649] | 0.87[0.82,0.92] | 0.39[0.31,0.47] | 0.33[0.23,0.43] |
| Pathologist 8 | 0.604[0.555,0.662] | 0.85[0.79,0.91] | 0.41[0.32,0.48] | 0.29[0.17,0.38] |
| Pathologist 9 | 0.760[0.707,0.809] | 0.86[0.80,0.91] | 0.64[0.57,0.71] | 0.53[0.40,0.63] |
| Pathologist 10 | 0.840[0.796,0.880] | 0.88[0.83,0.92] | 0.76[0.69,0.83] | 0.51[0.33,0.66] |
| Model | 0.970[0.941,0.998] | 0.94[0.88,0.97] | 0.93[0.89,0.97] | 0.79[0.65,0.90] |
| IM (slides) |  |  |  |  |
| Pathologist 1 | 0.738[0.684,0.787] | 0.69[0.60,0.77] | 0.61[0.53,0.68] | 0.46[0.27,0.61] |
| Pathologist 2 | 0.738[0.684,0.787] | 0.69[0.60,0.78] | 0.61[0.53,0.68] | 0.46[0.27,0.60] |
| Pathologist 3 | 0.738[0.684,0.791] | 0.69[0.60,0.78] | 0.61[0.53,0.68] | 0.46[0.29,0.61] |
| Pathologist 4 | 0.853[0.809,0.893] | 0.82[0.75,0.88] | 0.78[0.71,0.85] | 0.77[0.69,0.85] |
| Pathologist 5 | 0.764[0.716,0.818] | 0.65[0.57,0.74] | 0.65[0.57,0.73] | 0.45[0.29,0.61] |
| Pathologist 6 | 0.902[0.862,0.938] | 0.89[0.84,0.93] | 0.85[0.79,0.91] | 0.90[0.85,0.93] |
| Pathologist 7 | 0.867[0.818,0.907] | 0.86[0.78,0.91] | 0.80[0.74,0.87] | 0.84[0.78,0.90] |
| Pathologist 8 | 0.760[0.707,0.809] | 0.76[0.69,0.83] | 0.64[0.57,0.71] | 0.74[0.61,0.83] |
| Pathologist 9 | 0.853[0.809,0.898] | 0.80[0.74,0.88] | 0.78[0.72,0.85] | 0.81[0.71,0.88] |
| Pathologist 10 | 0.853[0.809,0.898] | 0.83[0.77,0.88] | 0.78[0.71,0.84] | 0.84[0.76,0.89] |
| Model | 0.949[0.918,0.972] | 0.81[0.74,0.88] | 0.81[0.75,0.87] | 0.81[0.69,0.89] |
| Atrophy (slides) |  |  |  |  |
| Pathologist 1 | 0.684[0.631,0.738] | 0.53[0.44,0.63] | 0.53[0.45,0.61] | 0.32[0.14,0.48] |
| Pathologist 2 | 0.684[0.635,0.738] | 0.54[0.45,0.64] | 0.53[0.45,0.60] | 0.33[0.15,0.49] |
| Pathologist 3 | 0.684[0.627,0.738] | 0.54[0.45,0.63] | 0.53[0.45,0.61] | 0.34[0.16,0.52] |
| Pathologist 4 | 0.649[0.600,0.702] | 0.66[0.58,0.74] | 0.47[0.39,0.55] | 0.43[0.31,0.54] |
| Pathologist 5 | 0.658[0.604,0.707] | 0.52[0.43,0.62] | 0.49[0.41,0.56] | 0.26[0.09,0.42] |
| Pathologist 6 | 0.800[0.751,0.853] | 0.64[0.55,0.72] | 0.70[0.62,0.77] | 0.62[0.48,0.73] |
| Pathologist 7 | 0.787[0.738,0.836] | 0.72[0.63,0.81] | 0.68[0.60,0.75] | 0.65[0.53,0.75] |
| Pathologist 8 | 0.582[0.529,0.631] | 0.66[0.58,0.74] | 0.37[0.29,0.45] | 0.31[0.20,0.42] |
| Pathologist 9 | 0.640[0.587,0.689] | 0.65[0.56,0.74] | 0.46[0.38,0.54] | 0.42[0.25,0.56] |
| Pathologist 10 | 0.742[0.689,0.791] | 0.58[0.50,0.68] | 0.61[0.53,0.69] | 0.35[0.17,0.50] |
| Model | 0.953[0.927,0.976] | 0.80[0.72,0.88] | 0.85[0.79,0.91] | 0.61[0.42,0.76] |
| OLGIM |  |  |  |  |
| Pathologist 1 | 0.688[0.583,0.792] | 0.51[0.33,0.73] | 0.50[0.30,0.67] | 0.65[0.36,0.82] |
| Pathologist 2 | 0.667[0.542,0.771] | 0.50[0.30,0.74] | 0.47[0.30,0.63] | 0.68[0.42,0.83] |
| Pathologist 3 | 0.667[0.562,0.771] | 0.50[0.30,0.73] | 0.47[0.30,0.63] | 0.68[0.44,0.83] |
| Pathologist 4 | 0.750[0.646,0.854] | 0.71[0.34,0.84] | 0.60[0.43,0.77] | 0.72[0.47,0.88] |
| Pathologist 5 | 0.833[0.729,0.917] | 0.81[0.69,0.93] | 0.73[0.57,0.87] | 0.88[0.75,0.96] |
| Pathologist 6 | 0.833[0.729,0.937] | 0.83[0.51,0.92] | 0.73[0.57,0.90] | 0.86[0.70,0.96] |
| Pathologist 7 | 0.812[0.708,0.896] | 0.75[0.48,0.92] | 0.70[0.53,0.87] | 0.88[0.78,0.95] |
| Pathologist 8 | 0.688[0.583,0.792] | 0.57[0.32,0.75] | 0.50[0.33,0.67] | 0.70[0.45,0.86] |
| Pathologist 9 | 0.812[0.708,0.917] | 0.71[0.50,0.88] | 0.70[0.53,0.87] | 0.84[0.66,0.94] |
| Pathologist 10 | 0.729[0.625,0.833] | 0.60[0.39,0.83] | 0.57[0.40,0.73] | 0.77[0.55,0.90] |
| Model | 0.792[0.688,0.896] | 0.71[0.55,0.87] | 0.67[0.50,0.83] | 0.78[0.54,0.92] |
| OLGA |  |  |  |  |
| Pathologist 1 | 0.562[0.458,0.667] | 0.37[0.16,0.60] | 0.30[0.13,0.47] | 0.34[-0.07,0.65] |
| Pathologist 2 | 0.583[0.479,0.688] | 0.40[0.20,0.62] | 0.33[0.17,0.50] | 0.40[0.02,0.68] |
| Pathologist 3 | 0.562[0.458,0.667] | 0.37[0.14,0.59] | 0.30[0.13,0.47] | 0.37[0.00,0.69] |
| Pathologist 4 | 0.542[0.438,0.646] | 0.26[0.09,0.48] | 0.27[0.13,0.43] | 0.15[-0.07,0.38] |
| Pathologist 5 | 0.708[0.604,0.812] | 0.56[0.39,0.78] | 0.53[0.37,0.70] | 0.59[0.25,0.83] |
| Pathologist 6 | 0.729[0.625,0.854] | 0.47[0.30,0.70] | 0.57[0.37,0.73] | 0.53[0.19,0.83] |
| Pathologist 7 | 0.792[0.688,0.896] | 0.73[0.54,0.88] | 0.67[0.50,0.83] | 0.73[0.43,0.92] |
| Pathologist 8 | 0.500[0.417,0.583] | 0.16[0.03,0.42] | 0.20[0.07,0.33] | 0.32[0.12,0.52] |
| Pathologist 9 | 0.646[0.542,0.771] | 0.56[0.21,0.75] | 0.43[0.27,0.63] | 0.19[-0.19,0.60] |
| Pathologist 10 | 0.562[0.458,0.667] | 0.26[0.11,0.47] | 0.30[0.13,0.47] | 0.30[0.03,0.54] |
| Model | 0.729[0.625,0.833] | 0.62[0.37,0.85] | 0.58[0.40,0.73] | 0.74[0.48,0.89] |

Table S6 The work experience of ten pathologists

|  | Work Experience (Year) |
| --- | --- |
| Pathologist 1 | 16 |
| Pathologist 2 | 12 |
| Pathologist 3 | 12 |
| Pathologist 4 | 9 |
| Pathologist 5 | 20 |
| Pathologist 6 | 6 |
| Pathologist 7 | 25 |
| Pathologist 8 | 9 |
| Pathologist 9 | 15 |
| Pathologist 10 | 13 |
